# Perceived stress in adolescence and labour market participation in young adulthood - a prospective cohort study

**DOI:** 10.1186/s12889-023-15120-0

**Published:** 2023-01-27

**Authors:** Trine Nøhr Winding, Mette Lykke Nielsen, Regine Grytnes

**Affiliations:** 1 Department of Occupational Medicine, University Research Clinic, Goedstrup Hospital, Møllegade 16, 1.st, Herning, 7400 Denmark; 2 Department of Culture & Learning, Centre for Youth Research, Aalborg University, Copenhagen, Denmark

**Keywords:** Perceived stress, Labour market participation, Adolescence, Cohort study, Life course epidemiology

## Abstract

**Background:**

Adolescence is a key-developmental stage for physical, neurological, psychological, and social changes. In this developmental stage, a large number of people struggle with mental health problems like stress, anxiety, or depression. Psychological vulnerability in adolescence has previously been found to be negatively related to future low labour market participation. However, studies are lacking that investigate the impact of stress during adolescence on labour market participation in early adulthood using register data. The aim of this prospective study was therefore to examine the association between perceived stress during adolescence and labour market participation in young men and women in early adulthood.

**Methods:**

A Danish cohort of 3038 participants born in 1989 was followed with use of questionnaires from age 15 to age 28. The exposure, self-reported perceived stress, was collected by questionnaires at ages 15, 18, and 21. The outcome, labour market participation, was based on register information on social benefits, such as unemployment benefits, sickness benefits, and disability benefits, collected on a weekly basis during a 4-year period. Information about the socioeconomic confounders was also gleaned from registers.

**Results:**

The study found consistent associations between perceived stress from age 15 to age 21 and low labour market participation from age 25 to age 29 in both women and men after adjusting for mental health and socioeconomic confounders. The strongest associations between perceived stress and low labour market participation were seen among men who reported stress several times during adolescence.

**Conclusions:**

The results indicate that although, women in general, reported being more stressed than men during adolescence and had lower labour market participation in early adulthood, there was a small group of men who had experienced stress during adolescence who were at particularly high risk of being marginalised in the labour market.

## Background

Labour market participation refers to the process in which people move from student to worker to join the labour force and gain positions in the occupational hierarchy [1]. In 2020, the general unemployment rate was 7.1% in Europe [2] and 4.6% in Denmark; however, for 25- to 29-year-old Danes, it was 8.2% [3]. An unstable attachment to the labour market among young adults is of rising concern due to its related negative social and health consequences as well as lack of sense of social identity [4–6]. Low labour market participation in early adulthood has been found to be associated with depressive symptoms independent of the type of participation (occupational status, employment status, job stability, earnings, and perceived career progress) [7], and if young adults are economically inactive, there is an increased risk of being economically inactive later in life [8]. Conversely, attachment to the labour market may improve future job prospects and prevent dependency on social benefits [8]. Therefore, it is crucial to study the risk factors of low labour market participation (LMP) in early adulthood in order to be able to address these factors and hence reduce individual and societal costs.

Adolescence is a key-developmental stage not only for physical, neurological, but also for psychological and social changes. In this developmental stage, a large number of people struggle with mental health problems like stress, anxiety, or depression [9–11]. Although young people from socioeconomic disadvantaged families or neighbourhoods are at increased risk of being stressed [12, 13], socioeconomic differences only explain a minor part of the increase in stress among young people in Denmark [14] and internationally [9–11]. Stress in young people is increasingly understood as a negative consequence of wider social trends in the “Performance Society” [15, 16], in which control over and success in life have become dominant ideals [17]. Young people generally behave extremely disciplined with regard to future choices and plans and direct their attention to the future and consider the consequences that their choices may have for their future possibilities [18], which can cause worry and stress [19] and eventually may lead to labour market marginalisation.

Previous studies show strong associations between the social circumstances present during adolescence, like low parental socioeconomic status [20, 21], parental divorce, disease, or abuse, and future reduced labour market participation [22]. Also, psychological vulnerability in adolescence like psychosomatic symptoms [23] has been shown to be associated with low labour market participation, whereas a study by Jensen et al. found that individual psychological resources such as high levels of mastery in adolescence, which is the feeling of control in life, opportunity to solve the problems that arise, and influence on one's own future, had a positive impact on labour market participation in early adulthood [24]. Mental health problems have likewise been found to relate to future labour market participation [25–27]. A recent Dutch longitudinal study found associations between mental health problems from age 11 to 26 and not having a paid job at age 26, measured by self-report [27]. Likewise, two studies by Egan et al. found that psychological distress at age 14 was associated with unemployment at ages 16 to 21 [28] and that poor mental health measured at one time point between ages 16 and 20 was associated with unemployment in the following 11-year period [29]. In both studies by Egan et al., exposure and outcome were based on self-report, and stress was measured at one time point. There is a lack of knowledge about the effect of persistent stress during adolescence on labour market participation in early adulthood. One study by Hanson et al. documented an association between accumulated stress occurring in childhood and the reward-related activity of the brain that led to reduced motivation and increased negative mood at age 26 [30]. However, it is unclear to what extent this biological stress response affects future work life.

Another aspect that needs further investigation is the potential gender difference in relation to stress and labour market participation. Young women typically report higher levels of perceived stress than young men [13, 31, 32], and there is evidence suggesting that women respond to and cope with stress differently than their male counterparts [13, 33]. However, conflicting results have been reported regarding gender differences in the association between various mental health aspects and labour market participation. A Swedish study found mental health conditions, defined in broad terms, at ages 18–19 to have long-term negative effects on future labour market participation in men [34]; however, this study did not include women. A Danish study found that men reporting somatic symptoms in late adolescence appeared to be more vulnerable to future reduced labour market participation than women [23]. However, only two identified studies have investigated the association between perceived stress and labour market participation for women and men separately and they both found the opposite gender trend compared to the abovementioned studies. A prospective study in the general Danish working population found a strong association between perceived stress measured at one time point and sickness absence in women but not in men [35] and, a study by Trolle et al. investigated the effects of perceived stress at age 21 on labour market participation 8 years later [31]. In the study higher levels of perceived stress were found to increase labour market participation in young men, whereas in young women, higher levels of perceived stress reduced future labour market participation [31]. Both exposure and outcome were measured for limited time periods in this study, which we wanted to improve in the present study.

In the present study stress is considered as a distinctive risk factor and understood as perceived or experienced stress, not as clinical stress. Lazarus' cognitive stress model [36] forms the basis for the definition and understanding of the phenomenon. An important element of Lazarus' theory is that the experience of stress is not static but develops in a dynamic interaction between the individual and the environment [37].

Altogether, the above points to the need to investigate whether perceived stress during adolescence leads to low labour market participation in men and women using register-based information about labour market participation in early adulthood.

### Aim

The aim of this study was to examine the association between perceived stress during adolescence (ages 15 to 21) and labour market participation in early adulthood (during a 4-year period from ages 25 to 29) in women and men.

### Hypothesis

Perceived stress at ages 15, 18 and 21 is associated with reduced labour market participation from age 25 to 29 in women and men.

## Methods

### Participants

The prospective study, The West Jutland Cohort, consisted of all individuals (*N* = 3681) born in 1989 and living in the county of Ringkjoebing, Denmark, in early April 2004. The West Jutland Cohort focuses on the study and exploration of different aspects of inequalities and social disparities and their effect on general health and well-being in a life course perspective [38, 39]. The initial questionnaire collection took place in 2004 at the schools within the county when the participants were 15 years old. Those not at school on the day of collection received the questionnaire by mail. Of the potential 3681 responders, 3054 (83%) adolescents participated (age 15). Data from two follow-up questionnaire rounds conducted by mail in 2007 (age 18) and 2010 (age 21), with response rates of 65% and 58% of the original source population, respectively, were also used. Further information on recruitment and data collection is given elsewhere [38]. The study population for the present study is comprised of participants who provided information about perceived stress on least one questionnaire and had available register information on labour market participation from ages 25 to 29 (*n* = 3038).

### Measures

Data comprised a combination of questionnaire and register data. In Denmark, every citizen is provided with a Civil Registration Number (CPR) at birth (or upon immigration). The CPR number allowed us to link questionnaire information to own and parental information from registers [40].

### Labour market participation

*Labour market participation* was defined as the degree of participation in education or employment from the age of 25 to the age of 29. Information about non-participation in education or lack of employment was obtained from the Danish Register for Evaluation and Marginalisation (DREAM) [41]. The DREAM register contains information on social benefits such as unemployment benefits, sickness benefits, and disability benefits on a weekly basis for all Danish citizens. Information about social benefits was collected from 1 January 2015 to 2 December 2018 (approximately 4 years), when the participants were 25- to 29-years old. Receipt of all types of social benefits was categorized as non-participation in education or lack of employment and classified as “passive LMP”, except maternity leave, educational grants, supplementary unemployment benefits, or not receiving any benefits at all, which were classified as “active LMP”. Based on the total number of weeks with passive LMP during the 4-year period, participants were dichotomised into “Low LMP” if they had received ≥ 12 months of passive LMP or “High LMP” if they had received < 12 months of passive LMP during the 4-year period.

### Perceived stress

To measure stress during adolescence, the abbreviated Perceived Stress Scale 4-item version (PSS-4) was used [36]. It is a global stress measure developed to assess the extent to which individuals find their lives to be unpredictable, uncontrollable, and overloaded. The four items were “In the last month, how often have you felt; 1) No influence on the essential things in your life?, 2) Confident about your ability to handle your personal problems?, 3) That things were going your way?, 4) Difficulties were piling up so high that you could not overcome them?”. Information was collected by questionnaires at ages 15, 18, and 21. The five response categories from (0) never to (4) very often were summarised to a scale ranging from 0 to 16 and dichotomised at the 75th percentile into high vs low stress score at ages 15 and 18 (≥ 7) and 21 (≥ 8). A measure of accumulated stress during adolescence was then generated containing the following three categories: 1. reported low stress scores in all three questionnaire rounds, 2. reported a high stress score in one of the three questionnaire rounds, 3. reported high stress scores in two or all three questionnaire rounds.

### Mental health

Mental health at age 15 was measured by an abbreviated 4-item version of the Center for Epidemiologic Studies Depression Scale for Children (CES-DC), which is designed to measure current levels of depressive symptoms in the general population [42]. The four items ask about one's mental state over the past week, with response categories from (0) not at all to (3) a lot, and were summarised to a scale ranging from 0 to 12 and dichotomised at the cut-off point of 3 and above, as recommended by Fendrich et al. [42].

### Socioeconomic measures

Parental socioeconomic position was defined by equivalised household income and highest attained parental level of education, both derived from Statistics Denmark. Both measures were included since the correlation between them was no more than *r* = 0.2. Equivalised household income was calculated from a yearly average of a 4-year period when the children were 7- to 10-years old, representing a period in the children’s lives where they were financially dependent on their parents. Equivalised income is a weighted average calculation divided by the number of household members based on a weight of 1.0 for the first adult, 0.5 for the second adult and persons above 14 years of age, and 0.3 for children below the age of 14 [43]. Income was then recoded into tertiles corresponding to low (< 11,072 €), medium (11,072–13,489 €), and high (> 13,489 €) yearly equivalised average income.

Highest attained parental level of education was obtained at age 15 (year 2004), and the participants’ own highest educational level was obtained at age 28 (year 2017) and in both cases categorised by primary level (< 10 years), secondary level (10–13 years), and tertiary level (> 13 years) of education [44].

Information about gender was based on register information [45]. All the register information was merged with questionnaire data by using information from the Central Office of Civil Registration [45].

### Statistical analysis

Regarding the perceived stress measures, the numbers of missing values were *n* = 726 (20%) at age 15, *n* = 1371 (37%) at age 18, and *n* = 1732 (47%) at age 21. Outcome information about labour market participation was missing for *n* = 12 (< 1%). Among covariates, the number of missing values ranged from *n =* 9 (< 1%) for own educational level to *n* = 681 (19%) for mental health. Initially, a multiple imputation chained model with 100 imputations was carried out to impute missing data for the three stress measures (from age 15 to age 21), the three measures of socioeconomic position, and the measure of mental health (age 15). Seven chains, regress, logit, or ologit, depending on the type of data, were constructed. Information on gender was complete. Besides the above-mentioned variables, some additional information about self-rated health was included when imputing the stress measures. The final estimates were found as the average of the *m* sets of estimates and the standard errors by applying a simple formula called Rubin’s rule (results not shown) [46].

A correlation analysis between all exposures and covariates showed the strongest correlations between perceived stress (age 15) and mental health (age 15), with a correlation of *r* = 0.53 and between perceived stress at age 15 and perceived stress at age 18, with a correlation of *r* = 0.40. All other correlations were below *r* = 0.32.

Descriptive statistics in relation to labour market participation were presented for women and men for all exposures and covariates as number and percentile distribution. Multiple logistic regression analyses were used to study the associations between perceived stress at ages 15, 18, 21 or accumulated adolescence stress and labour market participation from ages 25 to 29. First, the crude associations between adolescence perceived stress or covariates and labour market participation were calculated. Secondly, the associations between adolescence perceived stress and labour market participation were adjusted for own mental health and educational level and parental educational and income level. Covariates included in the analyses were chosen a priori based upon a systematic literature search.

Additionally, sensitivity analyses were conducted in which the cut-point for passive labour market participation was changed from 12 months to 24 months.

All odds ratios were presented with 95% confidence intervals (95% CIs) with a significance level of *p* < .05. Data analysis was performed by the statistical package Stata, statistical software version 16.0 (Stata Corporation, College Station, Texas, USA).

### Ethics

This study was conducted in accordance with the 1975 Declaration of Helsinki [47]. The study was approved by the Danish Data Protection Agency, which is an independent authority that supervises compliance with the rules on protection of personal data. The participants were informed about the purpose of the project, and their returning of the questionnaire was interpreted as informed consent and willingness to participate. This was in accordance with Danish regulations at the time of data collection. According to Danish law, questionnaire and register-based studies require neither approval by ethical or scientific committees nor written informed consent [48]. It is not possible to apply for ethical approval of studies unless they involved clinical examinations or biological material.

## Results

Table 1 shows the number and percentile distribution of exposures and covariates in relation to labour market participation in women and men. Of the final study population of 3038 participants, 50% were women, 16% of whom had low labour market participation at ages 25–29 compared to 11% of the men. The percentages of women reporting high perceived stress at ages 15, 18, and 21 were 34%, 33%, and 35% respectively. In men, the corresponding percentages were 22%, 21%, and 28%. Among women experiencing high perceived stress from age 15 to age 21, the percentages with low labour market participation increased from 18–24% with increasing age, compared to decreasing percentages between 15% and 11% among women with low perceived stress levels from age 15 to age 21. In men, experiencing high perceived stress from age 15 to age 21, between 16% and 18% of them had low labour market participation at ages 25 to 29 compared to percentages between 9% and 10% in men reporting low levels of stress from age 15 to age 21.

**Table 1:**
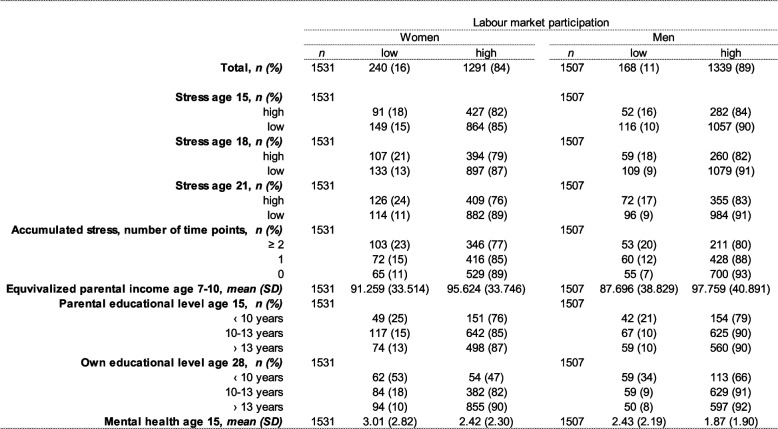
Distribution of exposure variables in relation to labour market participation at ages 25-29 by gender, *n* = 3038

The accumulated stress measure indicated that, among women and men experiencing stress at ≥ 2 time points between age 15 and age 21, 23% and 20% respectively had low labour market participation, compared to 11% and 7% among those that did not report feeling stressed between age 15 and age 21.

Results also show that among women with the highest educational level, 10% experienced low labour market participation compared to 53% among the lowest educated. Among the highest educated men, 8% experienced low labour market participation compared to 34% among the lowest educated. Likewise, the mean score of mental health problems was higher among those with low labour market participation than among those with high labour market participation, in both women and men.

Table 2 presents the crude and adjusted associations between perceived stress at ages 15, 18, and 21 and labour market participation at ages 25 to 29. I general, statistically significant crude and adjusted associations between perceived stress at the three time points and labour market participation were seen, except for perceived stress at age 15 and labour market participation, which was statistically non-significant in both genders after adjustment for potential confounders. The strength of the associations increased with increasing age, with men showing the strongest associations across age groups.

**Table 2:**
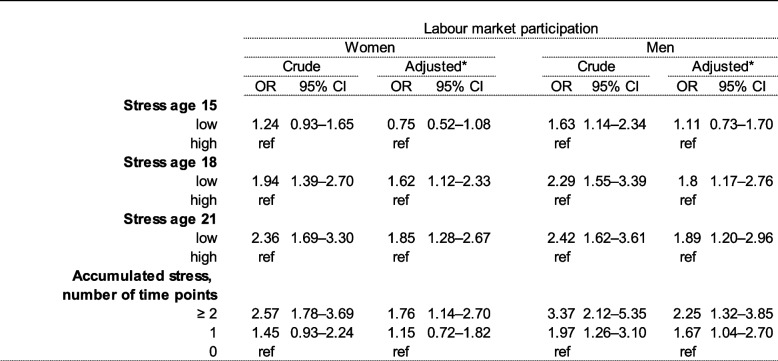
Assocations between stress at ages 15, 18 and 21 and labour market participation ages 25-29, *n* = 3038

^a^ Adjusted for equvivalized parental income, parental education level, own educational level, mental health

Clear exposure-response trends were seen across the three accumulated stress levels in both genders. Women having reported stress at a minimum of two out of three time points had a 1.76-fold statistically significant increased risk of low labour market participation in early adulthood, whereas the association for men was even higher, showing a 2.25-fold statistically significant increased risk after adjustment for potential confounders. In men, a statistically significant association was evident already when high perceived stress was reported at a single time point compared to not having reported feeling stressed (OR 1.67).

### Discussion

In the present study, we found consistent associations between perceived stress from age 15 to age 21 and low labour market participation from age 25 to age 29 in both women and men, which is in line with previous findings [28, 29]. As expected, a higher proportion of young women than young men experienced stress and low labour market participation [13, 31]. However, the strongest associations between perceived stress and low labour market participation were seen in men, which is consistent with the results from another Danish study investigating a different psychological issue, which showed that men reporting somatic symptoms in late adolescence were more vulnerable to future reduced labour market participation than women [23]. However, the results are contrary to the findings of the study by Trolle et al., where 21-year-old women having experienced stress had increased risk of low labour market participation 8 years later, but perceived stress in men reduced the risk of low labour market participation [31]. The reason for the conflicting results regarding gender is unclear. However, the study by Trolle et al. [31], measured perceived stress and mental health at only one time point, while perceived stress was measured at three time points from age 15 to age 21 in the present study, which may have had an impact on the results. In addition, Trolle et al. measured labour market participation during 1 year, while the present study examined it over a 4-year period. In the present study, we found that the negative effect on labour market participation was strongest in men when perceived stress was reported during three time points in adolescence. Likewise, a previous study by De Groot et al. found that a long duration of internalising problems increased the risk of not having a paid job in early adulthood [27].

The fight-or-flight response is generally regarded as the prototypic human response to stress, meaning that if a person meets a threat and determines that it has a realistic chance of being overcome, then “attack” is likely [49]. In circumstances in which the threat is perceived to be more insurmountable, flight is more probable. As such, an appropriate and modulated stress response is at the core of survival. However, in modern society it is not always possible to flee, and thus the general perceived stress level increases. It has been argued that “fight-or-flight” is the most typical respond to stress in men compared to females, who tend to respond in a more “tend-and-befriend” manner, where creating and maintaining social networks become the mode of survival [33]. These potential gender-specific reactions to stress could perhaps help to explain why men are particularly affected by stress of longer duration. The explanation would be that the “fight-or flight” strategy is efficient in order to deal with stress of shorter duration (be efficient and get things done), whereas when individuals are exposed to stress for a longer period of time, it causes stress overload which can ultimately lead to low labour market participation.

As already documented by others [20, 21], this study confirmed that a higher proportion of women and men from low socioeconomic conditions (especially in regard to own educational level) had low labour market participation compared to those from higher socioeconomic conditions. However, adjusting the associations between perceived stress and labour market participation for socioeconomic measures resulted in small attenuations but did not fully explain the associations. This indicates that the negative effect of perceived stress on future labour market participation is not solely explained by socioeconomic factors.

The increasing number of young people experiencing stress and other mental health problems early in life [9, 50] is worrying in relation to its potential negative effect on their future well-being and attachment to the labour market. In order to address and reverse increased stress in young people, we have to understand the pressure that is currently being put on young people to behave in a disciplined manner and make the correct choices for their future lives [18, 51]. This kind of performance pressure to succeed in life may lead to stress and mental health problems [15–17] which eventually can affect educational attainment and labour market participation [31]. Psychological vulnerability in adolescence manifested as psychosomatic symptoms, depression, and mental distress has previously been found to be negatively related to unemployment and future low labour market participation [23, 25, 28]. However, to our knowledge the present study is the first to investigate how perceived stress measured at several time points during adolescence affects gender-specific labour market participation from ages 25 to 29 using register data. The results show the importance of early stress detection and prevention to ensure young people a good and stable connection to the labour market later in life. There has previously been a strong focus on how the performance society stresses young women in particular. But the results of this study show that we must not forget the group of stressed young men because they are at particular risk of having an unstable labour market attachment already in early adulthood.

A sensitivity analysis changing the cut point of low labour market participation from 12 months to 24 months overall showed estimates pointing in the same direction, with a maximum increase in OR of 1.0 in women having experienced stress ≥ 2 previous time points (change in OR from 1.76 to 2.80).

### Strengths and limitations

Major strengths of this study are the use of objective register data with almost complete follow-up, the prospective design with a high initial response rate of 83%, and a 14-year follow-up period with perceived stress measures every third year from age 15 to age 21, which provides deep insight into the development of the changing impact of stress exposures during adolescence. According to Lazarus’s stress theory, the perceived level of stress is not a static condition but is influenced by daily hassles or major life events and may therefore change over time [37]. As perceived stress is a subjective assessment, the use of a self-reported questionnaire, the Perceived Stress Scale (PSS), is a reasonable choice. The PSS is designed to tap the degree to which respondents find their lives unpredictable, uncontrollable, and overloaded – central components of the experience of stress – and the measure takes the individual differences regarding the perception of stress into account [36].

However, some possible limitations of the present study should be considered. Regarding the 4-item perceived stress scale, it should be noted that because of the limited number of items in the PSS version used, the scale suffers in internal reliability and provides a less adequate approximation of the perceived stress levels compared to the 10-item and 14-item versions [36]. This could possibly introduce non-differentiated misclassification.

The social characteristics of the participants of The West Jutland Cohort study (*n* = 3681), such as the family's average disposable income and to some extent the parents' level of education, were in line with those of families of the same age throughout Denmark [13]. Thus, the participants are reasonably representative of young people nationwide. Comparison of the 643 non-responders to the 3038 responders regarding labour market participation and socioeconomic distribution shows that more non-responders (17%) than responders (13%) experienced low labour market participation and more non-responders had a low level of educational (27%) than did the responders (9%). The same tendency was seen in relation to parental socioeconomic distribution, however, less pronounced. This selection most likely lead to an underestimation of the true association and consequently bias away from the null hypothesis. The consequences of non-participation in connection with the first three questionnaire collection rounds are elucidated in a previous study, which found that despite the fact that non-responders are more likely to come from homes with a lower educational and income level, it had no significant impact on the validity of the measured risk estimates [38]. The present study consisted of participants who had answered the questions about perceived stress from least one questionnaire round and with register information on labour market participation from ages 25 to 29 (*n* = 3038). Multiple imputation analysis was conducted, which allowed for an analysis of the total sample, potentially adding to statistical power and diminishing bias [52]. However, the external validity and generalisability of the present study are limited to people in Denmark and countries with similar welfare systems. Even within welfare societies differences in social conditions can exist, which can have an impact on stress in men as well as women.

The use of self-reported questionnaire information entails the risk of participants' over- or under-reporting, and the risk that a participant's negative response to a question related to well-being in one question may tend to negatively affect their answers to other questions related to well-being or mental health. However, the use of both questionnaire and register data as well as prospectively collected data decreased this potential bias, called common method bias [53]. Although the presented limitations are not considered to cause serious selection or information bias in relation to the observed associations, caution about a causal interpretation is warranted.

## Conclusions

A strong association between perceived stress from age 15 to age 21 and low labour market participation in early adulthood has been documented, especially among men having experienced accumulated stress during adolescence. Although more women report being stressed during adolescence and consequently have low labour market participation in early adulthood, we have identified a small group of men who experience stress at several points during adolescence and consequently are at particularly high risk of future low labour market participation. To address the challenges in this group, it is important to examine further what is at stake in this group in order to avoid marginalisation from the labour market.

## Data Availability

The data that support the findings of this study are available from Statistics Denmark but restrictions apply to the availability of these data, which were used under license for the current study, and so are not publicly available. Data are however available from the authors upon reasonable request and with permission of the corresponding author (TNW).
